# Atypical presentation of dopa‐responsive dystonia in Taiwan

**DOI:** 10.1002/brb3.906

**Published:** 2018-01-20

**Authors:** Yi Ching Weng, Chun Chieh Wang, Yih Ru Wu

**Affiliations:** ^1^ Department of Neurology Chang Gung Memorial Hospital Chang‐Gung University College of Medicine Taipei Taiwan

**Keywords:** atypical presentation, dopa‐responsive dystonia, onset age, parkinsonian, Segawa disease

## Abstract

The typical clinical presentation of dopa‐responsive dystonia, which is also called Segawa disease, is a young age of onset, with predominance in females, diurnal fluctuation of lower limb dystonia, and fair response to low‐dose levodopa. This disease has both autosomal dominant and autosomal recessive inheritance. Autosomal dominant Segawa disease is caused by *GCH1* mutation on chromosome 14q22.1‐q22.2. Here, we report the case of a male patient with genetically confirmed Segawa disease and atypical presentations including no diurnal symptom fluctuation and insufficient response to levodopa. The patient's father who had the same mutation presented parkinsonism in old age. We also review the literature to address the broad clinical heterogeneity of Segawa disease and the influence of onset age on clinical presentation.

## 1 INTRODUCTION

Segawa disease, also called dopa‐responsive dystonia (DRD), is an autosomal inherited disease classified as a dystonia‐plus syndrome; it was first described by Segawa, Ohmi, Itoh, Aoyama, and Hayakawa (1971) as a disease with marked diurnal fluctuation of dystonia. Segawa disease is characterized by a typical clinical presentation of young‐onset lower limb dystonia (Van Hove et al., 2006) and the dystonia becomes more severe near the end of the day (Nygaard, Marsden, & Duvoisin, 1988). Another characteristic is that the dystonia favorably responds to low‐dose levodopa (Nygaard et al., 1988). The prevalence of this disease is approximately 0.5–1 per million (Segawa, Nomura, & Nishiyama, 2003; Tg, 1993; Zirn et al., 2008) and the data could be highly under‐representative due to mild symptoms in some patients; because of a higher penetrance of the causative genes among females, the incidence of Segawa disease is 2–4 higher in females than in males (Furukawa et al., 1998; Steinberger et al., 1998). With time, the lower limb dystonia progresses to generalized dystonia (Segawa et al., 2003).

Segawa disease is caused by to a mutation in the guanosine triphosphate cyclohydrolase 1 gene (*GCH1*) on chromosome 14q22.1‐q22.2 (Ichinose et al., 1994; Nygaard et al., 1993; Segawa et al., 2003). GCH1 is the rate‐limiting enzyme in tetrahydrobiopterin (BH4) synthesis, and BH4 is a cofactor for tyrosine hydroxylase (Ichinose et al., 1994). Patients with *GCH1* mutations demonstrated reduced GCH1 activity, thus leading to decreased dopamine levels and resulting in dystonia (Nagatsu, Levitt, & Udenfriend, 1964). However, atypical presentations of Segawa disease, such as that with incomplete response to levodopa (Bandmann et al., 1996; Regula, Thoden, & Meinck, 2007; Romstad et al., 2003) and presentation as cerebral palsy, spastic paraplegia, pure parkinsonism, or proximal weakness and hypotonia (Bandmann, Marsden, & Wood, 1998; Bandmann et al., 1996; Fink et al., 1988; Kong, Ko, Tong, & Lam, 2001; Nygaard et al., 1988), have been reported in a relatively few cases. Here, we report a rare case of Segawa disease with an atypical presentation and provide a literature review addressing the clinical heterogeneity of Segawa disease.

## 2 CASE REPORT

**Figure 1:**
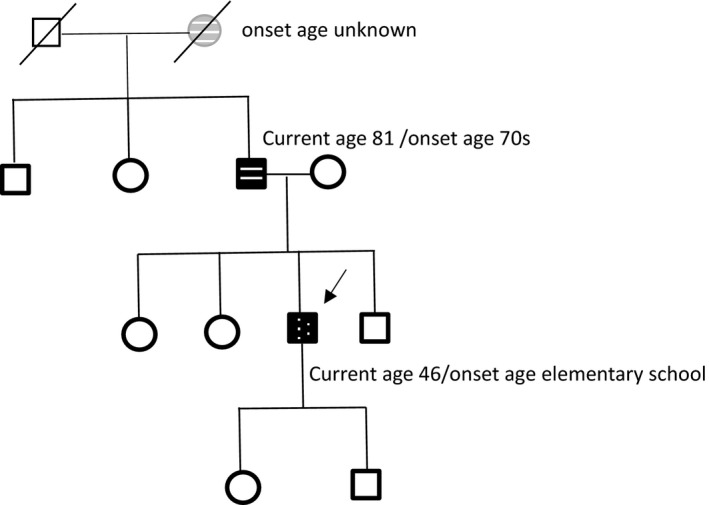
Pedigree of the study family. A genetic study was performed for the index case (arrow) and his father. Black indicates positive clinical signs; gray indicates suspicious clinical symptoms. Patient with parkinsosian symptoms was indicated with horizontal line symbol and patient with dystonia symptoms was indicated with dotted symbol

**Figure 2:**
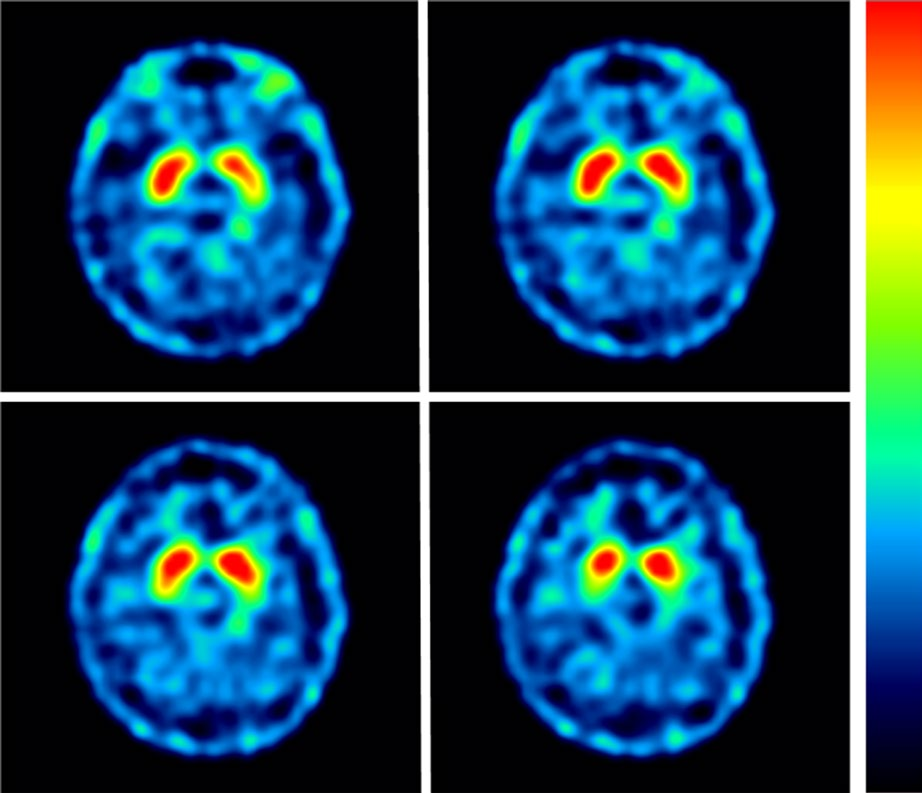
Image of 99mTc‐TRODAT‐1 SPECT of index patient. The image revealed bilateral symmetry of the normally shaped caudate nucleus and putamen. Symmetrical distribution of dopaminergic radioactivity was noted in the striatum. The result revealed no presynaptic uptake decreasing

**Figure 3:**
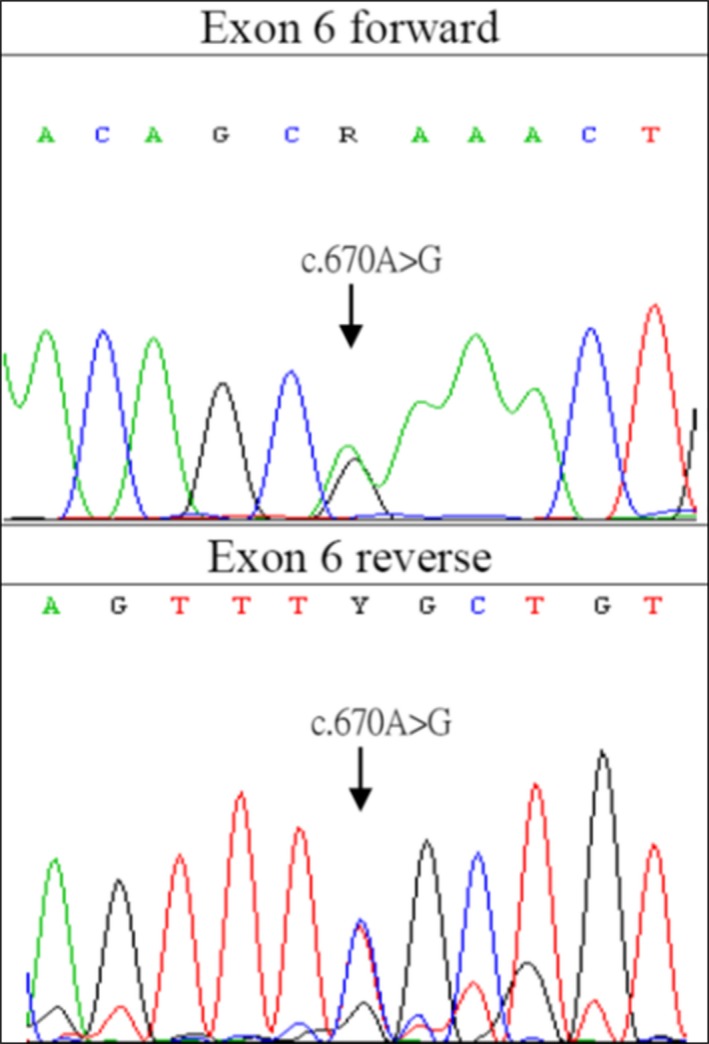
Genetic study of the patient. A mutation was noted at exon 6 c.670 A>G

A 43‐year‐old man, without underlying diseases, had left‐foot dystonia for 3 years since 2011. The symptoms had progressed for 2 months before he sought medical help. Because of dystonic movement, he could not walk normally on flat ground; nevertheless, he could walk backward, run, and climb upstairs and downstairs without difficulty. No diurnal fluctuation of his left‐foot dystonia was noted. The birth and developmental histories were normal for this patient. His personal, trauma, and drug exposure histories were also unremarkable. However, in elementary school, he was informed that he had a mildly abnormal walking stance, although this did not impair his daily function. His father was diagnosed of Parkinson' s disease since his 70s with the clinical presentations of rest tremor, bradykinesia and rigidity on the left side and then festinating gait. These symptoms responded to levodopa treatment. He had regullarily follow‐up in another medical center in Taipei and we had examined and interviewed him once in our outpatient clinic. He also had REM sleep behavior disorder symptom. The patient's grandmother also had parkinsonian features according to patient's statement. Other family members are unremarkable (Figure 1). Extensive neurological examination of the patient did not reveal any significant abnormality including parkinsonian signs, except for inversion of the left ankle with dorsiflexion of the big toe when the patient walked on the ground. No Kayser–Fleischer ring or parkinsonian symptom was noted. His medication including trihexyphenidyl up to 4 mg and clonazepam 0.5 mg was ineffective. He was also administered levodopa with DTI (decarboxylase inhibitor) 100 mg per day, which alleviated his symptoms for only the first 10 days. However, thereafter, even after increasing the dose to 300 mg per day, the patient subjectively felt no response; thus, he stopped medication use. His laboratory survey results including ceruloplasmin, blood and urine copper levels and thyroid, liver, and renal function were normal. The results of imaging studies including brain MRI and ^99m^Tc‐TRODAT‐1 SPECT were also unremarkable (Figure 2). For further diagnosis of dystonia, we conducted a genetic study; the results confirmed Segawa disease with *GCH1* mutation at exon 6 c.670 A>G(p.Lys224Glu) (Figure 3). We only conducted genetic study of *DJ1, ATP13A2, PINK1, UCHL1, SNCA, PARKIN,* and *LRRK2* using multiplex ligation‐dependent probe amplification (MLPA) assay for Parkinson's disease due to pure dystonia symptom for long time without parkinsonian symptom and normal TRODAT study. There was no copy number variation in these genes. Genetic testing for dystonia‐causing genes such as Tor1A, TH, or PRKRA was not conducted as the subject's presentation was not consistent with the phenotype reported for each of these mutations. Because of the parkinsonian presentation observed in his father and possibility of inheritance, we arranged a genetic study for his father; he was also confirmed to have the same gene mutation at exon 6 c.670 A>G. The patient's grandmother had already passed away; therefore, no further genetic study could be performed. We further conducted genetic study of c.670 in a cohort of 173 normal controls with 346 chromosomes, and no c.670 A>G was found. Moreover, because the patient did not have fair response to levodopa, we performed botulinum injection over his anterior tibialis, tibialis posterior, and extensor hallucis longus muscles. Consequently, his dystonia improved considerably from Global Dystonia Rating Scale (GDS) 5 to 1. The patient was satisfied with the treatment results.

## 3 DISCUSSION

We report the case of an atypical presentation of Segawa disease including insufficient drug response and no diurnal fluctuation of dystonia. A good response to low‐dose levodopa is considered characteristic for the diagnosis of Segawa disease (Müller, Steinberger, & Topka, 2002; Nygaard et al., 1988). Diurnal fluctuation is often present in the affected patients (Nygaard et al., 1988) and the rate of this fluctuation is approximately 94% (Liu et al., 2010). After searching PubMed for reports on Segawa disease, we found only a few reports mentioning poor or incomplete levodopa response (Bandmann et al., 1996; Grimes, Barclay, Duff, Furukawa, & Lang, 2002; Regula et al., 2007; Romstad et al., 2003; Tadic et al., 2012; Tassin et al., 2000). One patient had tyrosine hydroxylase deficiency and other cases had *GCH1* mutation. According to the previous reports, a daily levodopa dose of <20 mg/kg is sufficient for treating Segawa disease without a DTI (Segawa et al., 2003) whereas with a DTI, a daily dose of 300 mg, and sometimes 400 mg, is required (Nygaard, Snow, & Fahn, 1993; Steinberger et al., 2000). Although the ineffectiveness of levodopa may be explained by the extent of its decarboxylation in the intestines, patients with Segawa disease having insufficient drug response have rarely been reported thus far. In our patient, both the absence of diurnal fluctuation of dystonia and insufficient levodopa response rendered the diagnosis of Segawa disease increasingly difficult.

Moreover, the clinical course of Segawa disease is onset age‐dependent (Segawa, Hosaka, Miyagawa, Nomura, & Imai, 2000). In Trender‐Gerhard's 2008 cohort study (Trender‐Gerhard et al., 2009) 34 patients with Segawa disease were divided into four groups according to their clinical presentation: young‐onset classic (onset age, 4.5 ± 4.6 years), young‐onset mild (onset age, 7.3 ± 3.9 years), young onset with severe initial hypotonia (onset age, 0.3 ± 0.3 years), and adult onset (onset age, 37.0 ± 8.2 years). In the young‐onset classic group, the female‐to‐male ratio was 2.3:1, a normal ratio for Segawa disease. However, in the young‐onset mild and adult‐onset groups, male predominance was noted (female:male = 1:3). This result reconsiders the basic concept of female predominance in Segawa disease. In addition, in the young‐onset mild group (Trender‐Gerhard et al., 2009) the patients' symptoms were mild, not even requiring treatment. In our case, the patient had mild symptoms and had no severe progression until adulthood; this presentation is similar to that reported in the literature regarding gender as well as clinical presentation (Trender‐Gerhard et al., 2009). Furthermore, tremors or parkinsonism can be the main or only sign in adult‐onset patients (Segawa et al., 2003). In the adult‐onset group of Trender‐Gerhard's study, all the adult patients presented tremors or parkinsonism as main symptoms. In our case, the patient's father was confirmed to have *GCH1* mutation with typical parkinsonian features, which is also similar to the report in the previous study (Segawa et al., 2003; Trender‐Gerhard et al., 2009). A similar classification has been summarized previously (Bernal‐Pacheco et al., 2013). According to the literature and our observations, we conclude that mild symptoms of Segawa disease tend to occur in young‐onset male patients and that pure parkinsonism could be a common feature in adult‐onset male patients with *CGH1* mutation.

For the completely different clinical presentation of Segawa disease in our report, we further searched Pubmed with the keywords “Parkinsonism” and “Segawa” or “DRD,” and we found a few familial study articles on Segawa disease with symptoms of both dystonia and parkinsonism that are similar to those in our familial study (Table 1). In these families, adult‐onset cases apparently tended to present pure parkinsonism (Table 2); in addition, the number of adult‐onset male patients was higher than that of female patients (Table 2). Young‐onset patients could have more varied presentation but still predominantly had pure dystonia or parkinsonism with dystonia (Table 3). In the meta‐analysis study of Tadic et al., adult‐onset patients could have more parkinsonian presentation than young‐onset patients and this result is also not against our finding (Tadic et al., 2012). In a recent study of Mencacci et al. (2014) they found *GCH1* gene could possibly have a correlation with Parkinson's disease via whole exome sequencing;. Another study of *GCH1* single‐nucleotide polymorphisms revealed possible increased risk of Parkinson's disease (Chen et al., 2016). Whether *GCH1* mutation could be pathogenic to Parkinson's disease remained uncertain, but these results could possibly give us a new idea that parkinsonism may not necessary just be symptoms of DRD.

**Table 1:** Summary of reports on familial studies of Segawa disease with confirmed *GCH1* mutations

Author	Published year	Family members with *GCH1* gene mutation	Pure parkinsonism sign, patient onset age and sex	Dystonia with or without parkinsonism sign, patient onset age and sex	*GCH1* mutation location
Tassin et al. (2000)	2000	5	57 (F), 55 (M)	10 (M), 10 (M),13 (M)	c.538C>T Gln180stop
Tassin et al. (2000)	2000	6	52 (M)	10 (F), 10 (F), 11 (M), 18 (F), 42 (F)	IVS5+1G>A
Tassin et al. (2000)	2000	2	12 (F)	8 (F)	631‐632delAT
Grimes et al. (2002)	2002	10	56 (F)	1 (F), 1–2 (F), 2 (F), 3 (M), childhood (M)	18 base pair deletion in exon 1
Romstad et al. (2003)	2003	18	65 (M), 41 (M), 15 (M), 10 (M), 6 (M), 6 (M)	9 (F), 6 (M), 3 (M), 2.5 (M)	c.899C>T
Antonino Uncini et al.	2004	16	50 (M), 46 (M)	Childhood (M), childhood (M), childhood (M), childhood (F), <6 (M), <6 (F), 5 (F), 4 (F), birth (M)	5‐base pair insertion at codon 243
Eggers C et al.	2012	2	50 (F)	10s (F)	Complete deletion of the *GCH1* gene on one allele
Bernal‐Pacheco et al. (2013)	2013	6	41 (F), 40 (F), 31 (F)	7 (F), 7 (F), 6 (F)	c.159delG
Mencacci et al. (2014)	2014	3	59 (M)	1.5 (M)	c.343+5G>C
Mencacci et al. (2014)	2014	2	66 (M)	4 (F)	c.610G>A, c.722G>A
Mencacci et al. (2014)	2014	2	44 (F)	Childhood (F)	c.626+1G>C
A.J. Lewthwaite et al.	2015	5	58 (M), 50 (M)	44 (M), 17 (F), 6 (F)	c.5A>G

Cases within each family had either pure parkinsonism or dystonia with or without parkinsonism. Patients with uncertain sign or onset age were excluded.

**Table 2:** Further evaluation of adult‐onset cases with pure parkinsonism or dystonia symptom according to gender

Adult‐onset Segawa disease	Pure parkinsonism	Dystonia with or without parkinsonism
Male	10	1
Female	7	1

An obvious trend for adult‐onset patients to present pure parkinsonism was noted.

**Table 3:** Further evaluation of young‐onset (below 18 years old) cases with pure parkinsonism or dystonia symptoms according to gender

Young‐onset Segawa disease	Pure parkinsonism	Dystonia with or without parkinsonism
Male	4	15
Female	1	20

Until 2016, 229 mutations were collected in the Human Gene Mutation Database (http://www.hgmd.org/). The *GCH1* mutation at exon 6 c.670 noted in our patient has been reported previously in patients with DRD (Bandmann et al., 1996; Jarman, Bandmann, Marsden, & Wood, 1997; Mencacci et al., 2014). Although this is not the first report regarding this mutation location, the previous reports reported either lysine to arginine or lysine to threonine change and cause conservative amino acid change or stop codon, whereas we discovered a lysine to glucine change in our case (Bandmann et al., 1996; Jarman et al., 1997). We also confirmed that lysine to glucine change is not a variant but a mutation in our study. The clinical presentations of each patient are different, and the mechanisms of this mutation to cause various clinical features are still not well understood. We reviewed a previous report and found that Arg249Ser, Ser81Pro, Ser76X, Gly203Arg, 249del A, and IVS5+3insT have been reported in Taiwan (Hwu, Chiou, Lai, & Lee, 2000; Wu‐Chou et al., 2010). In a group of Chinese Han patients, the locus included Gly155Ser in exon 3 (Hu et al., 2011); Met137Arg in exon 2 (Hu et al., 2011); Gly203Arg in exon 5 (Hu et al., 2011; Liu et al., 2010; Yu, Zhou, Hu, & Xu, 2013); Gln161Pro (Xie et al., 2006); Lys224Arg (Xie et al., 2006); Met1Thr (Cao et al., 2010); Ser80Asn (Cao et al., 2010); Leu82Pro (Cao et al., 2010); and IVS5+3 del AAGT (Cao et al., 2010); Met102Lys (Liu et al., 2010); Thr186Ile (Liu et al., 2010); Tyr75Cys (Cai et al., 2013); Ala98Val (Cai et al., 2013); Ile135Thr (Cai et al., 2013); Leu91Val (Wu et al., 2008); Pro95Leu (Wu et al., 2008); Val204Gly (Wu et al., 2008) and 628delC (Wu et al., 2008); Met1Ile (Li et al., 2007); Thre94Met (Yu et al., 2013); Leu145Phe (Yu et al., 2013); and c. 453+6G>T (Yu et al., 2013). A study of 40 patients with Segawa disease suggested that most mutations in Chinese patients with Segawa disease are clustered in two regions—the 210–360 and 550–650 base pair (Yu et al., 2013).

In conclusion, we report a case with an atypical presentation of Segawa disease and aim to address the broad clinical heterogeneity of Segawa disease.

## CONFLICT OF INTEREST

None of the authors has any conflict of interest to disclose.
