# Supplementary material for: Atypical presentation of dopa‐responsive dystonia in Taiwan
Source: Brain Behav. 2018 Jan 20;8(2):e00906. doi: 10.1002/brb3.906 (PMC5822572; doi:10.1002/brb3.906)
Supplement: Supplementary file 1 [file BRB3-8-e00906-s001.docx]

Uncini, A., De Angelis, M.V., Di Fulvio, P., Ragno, M., Annesi, G., Filla, A., ... Gambi, D. (2004). Wide expressivity variation and high but no gender-related penetrance in two dopa-responsive dystonia families with a novel GCH-I mutation. ***Movement Disorders****,* 19, 1139-1145.

Eggers, C., Volk, A. E., Kahraman, D., Fink, G. R., Leube, B., Schmidt, M., Timmermann, L. (2012). Are Dopa-responsive dystonia and Parkinson's disease related disorders? A case report. ***Parkinsonism & Related Disorders****,* 18, 666-668.

Lewthwaite, A. J., Lambert, T. D., Rolfe, E. B., Olgiati, S., Quadri, M., Simons, E. J., ... Niccoll, D. J. (2015). Novel GCH1 variant in Dopa-responsive dystonia and Parkinson's disease. ***Parkinsonism & Related Disorders****,* 21, 394-397.
